# Genomic Responses during Acute Human Anaphylaxis Are Characterized by Upregulation of Innate Inflammatory Gene Networks

**DOI:** 10.1371/journal.pone.0101409

**Published:** 2014-07-01

**Authors:** Shelley F. Stone, Anthony Bosco, Anya Jones, Claire L. Cotterell, Pauline E. van Eeden, Glenn Arendts, Daniel M. Fatovich, Simon G. A. Brown

**Affiliations:** 1 Centre for Clinical Research in Emergency Medicine, Harry Perkins Institute of Medical Research and the University of Western Australia, Perth, Australia; 2 Department of Emergency Medicine, Royal Perth Hospital, Perth, Australia; 3 Telethon Kids Institute and the Centre for Child Health Research, University of Western Australia, Perth, Australia; Cincinnati Children's Hospital Medical Center, University of Cincinnati College of Medicine, United States of America

## Abstract

**Background:**

Systemic spread of immune activation and mediator release is required for the development of anaphylaxis in humans. We hypothesized that peripheral blood leukocyte (PBL) activation plays a key role.

**Objective:**

To characterize PBL genomic responses during acute anaphylaxis.

**Methods:**

PBL samples were collected at three timepoints from six patients presenting to the Emergency Department (ED) with acute anaphylaxis and six healthy controls. Gene expression patterns were profiled on microarrays, differentially expressed genes were identified, and network analysis was employed to explore underlying mechanisms.

**Results:**

Patients presented with moderately severe anaphylaxis after oral aspirin (2), peanut (2), bee sting (1) and unknown cause (1). Two genes were differentially expressed in patients compared to controls at ED arrival, 67 genes at 1 hour post-arrival and 2,801 genes at 3 hours post-arrival. Network analysis demonstrated that three inflammatory modules were upregulated during anaphylaxis. Notably, these modules contained multiple hub genes, which are known to play a central role in the regulation of innate inflammatory responses. Bioinformatics analyses showed that the data were enriched for LPS-like and TNF activation signatures.

**Conclusion:**

PBL genomic responses during human anaphylaxis are characterized by dynamic expression of innate inflammatory modules. Upregulation of these modules was observed in patients with different reaction triggers. Our findings indicate a role for innate immune pathways in the pathogenesis of human anaphylaxis, and the hub genes identified in this study represent logical candidates for follow-up studies.

## Introduction

Anaphylaxis is a severe allergic reaction affecting multiple organ systems, characterized by generalized erythema-urticaria, plus cardiovascular compromise (hypotension) and/or respiratory features (breathlessness, bronchospasm and hypoxemia). Foods, insect stings and drugs cause roughly equal proportions of reactions. Allergen crosslinking of allergen-specific IgE bound by high affinity (FcεRI) receptors to mast cells in the gut, skin and perivascular tissues including coronary vessels is the predominant triggering mechanism. An array of preformed and newly synthesized biochemical mediators with overlapping biological effects are then released [1]. However, the mechanism by which minute amounts of allergen administered locally (e.g. a sting to the skin, or minute amount of ingested food) leads to massive levels of systemic mediator release and death within minutes of exposure is not fully understood [2].

Several groups of immune mediators have independent associations with reaction severity, suggesting a synergistic involvement of multiple inflammatory pathways in human anaphylaxis [3]. Possible amplification mechanisms include mediators from triggered mast cells having a direct effect on other mast cells [4], and the involvement of other immune cells, including peripheral blood leukocytes (PBL). The concept of a “mast cell-leukocyte cytokine cascade” has been proposed in the context of allergic airway inflammation [5], and neutrophils and basophils have been found to have pivotal roles in mouse models of anaphylaxis [6], [7]. However, mouse models are largely IgG-mediated and there is no evidence for the involvement of circulating leukocytes in human anaphylaxis.

We therefore aimed to improve our understanding of the pathophysiology of human anaphylaxis by investigating gene expression patterns in PBL collected during anaphylaxis.

## Methods

### Study population

Patients were recruited in the Royal Perth Hospital ED as part of the Critical Illness and Shock Study [8]. Because the need for emergency care took priority, waiver of initial consent was approved under the provision of paragraph 2.3.6 of the National Health and Medical Research Council Ethical Conduct guidelines (2007). Once treatment was started, fully informed written consent was obtained as soon as possible and patients were given the option of declining further involvement and having all research samples collected up to that point destroyed. Ethics approval, including waiver of initial consent, was obtained from the Royal Perth Hospital Human Research Ethics Committee (EC 2009/080).

We enrolled a convenience sample of six consecutive adult patients, presenting when a research nurse was on duty with typical anaphylaxis according to the National Institutes Allergy and Infections Diseases/Food Allergy and Anaphylaxis Network definition of anaphylaxis [9], and who had not received any treatment prior to ED arrival. A structured datasheet was used to record demographics, reaction features, likely causation (if known), co-morbidities, physiological observations and treatments. Reaction severity was graded according to our established grading system [10]. Samples were also collected from six age-sex matched healthy controls with no history of anaphylaxis.

### Sample collection and storage

Blood samples were collected at enrolment (arrival in the ED), 1 hour and 3 hours after enrolment in both patients and controls, and stabilized in PAXgene tubes (PreAnalytiX GmbH, Switzerland). The PAXgene tubes were placed at 4°C then transferred to −20°C within 72 hours, before final storage at −80°C.

### RNA extraction

RNA was extracted with the PAXgene Blood RNA Extraction Kits (PreAnalytiX GmbH, Switzerland) by automation with the Qiacube instrument (Qiagen, Australia). The purity and integrity of the RNA was assessed on a NanoDrop (Thermo Scientific, Australia) and Bioanalyzer (Agilent), and was very high (median OD 260/280 ratio of 2.1 (IQR 2.06–2.14); median RIN 8.4 (IQR 8.1–8.8)).

### Gene expression profiling

Total RNA samples were labeled and hybridized to Affymetrix Human Gene 1.0 ST microarrays at the Ramaciotti Centre for Gene Function Analysis (University of New South Wales). The microarray data was high quality (mean±sd; pm mean  = 454±89; all probeset mean  = 6.62±0.02; pos vs neg auc  = 0.83±0.01; mad residual mean  = 0.35±0.04; relative log expression mean  = 0.2±0.04). The raw microarray data are available from the Gene Expression Omnibus repository (accession number GSE47655).

The microarray data was analyzed in the R environment for statistical computing. The data was preprocessed employing the Factor Analysis for Robust Microarray Summarization algorithm (qFARMS; laplacian prior was used) [11]. A custom chip description file was used to map probe sets to genes based on current annotation of the genome (hugene10sthsentrezg; version 16) [12]. The informative/non-informative calls algorithm was employed to identify relevant gene expression signals and filter out noise [13]. The final filtered data set comprised 5,292 genes and 36 samples, and this filtered data set was used for all downstream analyses.

Differentially expressed genes were identified using Bayesian/moderated *t*-statistics (LIMMA), and those genes with a False Discovery Rate (FDR) adjusted *p*-value of less than 0.05 were deemed significant [14]. Molecular signatures from the Molecular Signatures Database (http://www.broadinstitute.org/gsea/msigdb/index.jsp) were tested for differential expression employing Gene Set Analysis, with FDR control for multiple testing [15]. A coexpression network was constructed employing weighted gene coexpression network analysis (WGCNA) [16]–[18]. Modules of coexpressed genes were tested for differential expression in anaphylaxis cases versus controls employing Correlation Adjusted MEan RAnk gene set analysis (CAMERA) [19]. The wiring diagram of the disease-associated modules was reconstructed in Ingenuity Systems software using mechanistic data from prior studies [18]. Genes with no previously documented molecular interactions were removed from the analysis. Biological functions and pathways enriched in the data were identified using the database for annotation, visualization and integrated discovery (DAVID) [20]. Additional pathways analyses were performed with Ingenuity Systems software. Ingenuity Systems Upstream Regulator analysis was employed to infer putative driver genes or drugs/compounds that may give rise to the observed gene expression changes, and an overlap p-value was calculated based on the number of differentially expressed genes identified in the data that are known to be regulated by the upstream regulator.

## Results

### Patients

Details for each patient are presented in Table 1. Reactions were of moderate severity (i.e. without hypotension or hypoxemia) at the time of ED arrival. Reactions satisfied consensus clinical criteria for a diagnosis of anaphylaxis [9], and were attributed to aspirin (n = 2), peanut (n = 2), bee sting (n = 1) and unknown cause (n = 1). All patients were untreated at T0 and were treated with combinations of steroids, intravenous (IV) fluids and epinephrine between T0 and T1.

**Table 1:** Reaction features.

ID	Age/Sex	Time (mins)*	Treatment	Cause	Symptoms
10299	36F	90	Steroids IV fluid	Aspirin	Generalized urticaria, periorbital edema, angioedema, dyspnea, dizziness, chest/throat tightness, marked tachycardia
10325	21M	90	Steroids IV fluids epinephrine	Aspirin	Periorbital edema, wheeze, chest/throat tightness
10331	22F	125	Steroids IV fluids epinephrine	Peanut	Generalized urticaria, angioedema, dyspnea, chest/throat tightness, abdominal pain
10391	57F	90	Steroids epinephrine	Peanut	Generalized urticaria and erythema, periorbital edema, wheeze, nausea
10129	31M	48	Steroids IV fluids epinephrine	Bee	Generalized urticaria and erythema, angioedema, dyspnea, wheeze, chest/throat tightness
10188	18M	45	IV fluid epinephrine	Unknown	Periorbital edema, wheeze, chest/throat tightness

*Time from illness onset to first blood sample collected in the ED.

### Gene expression profiling of acute anaphylaxis in whole blood

**Figure 1:**
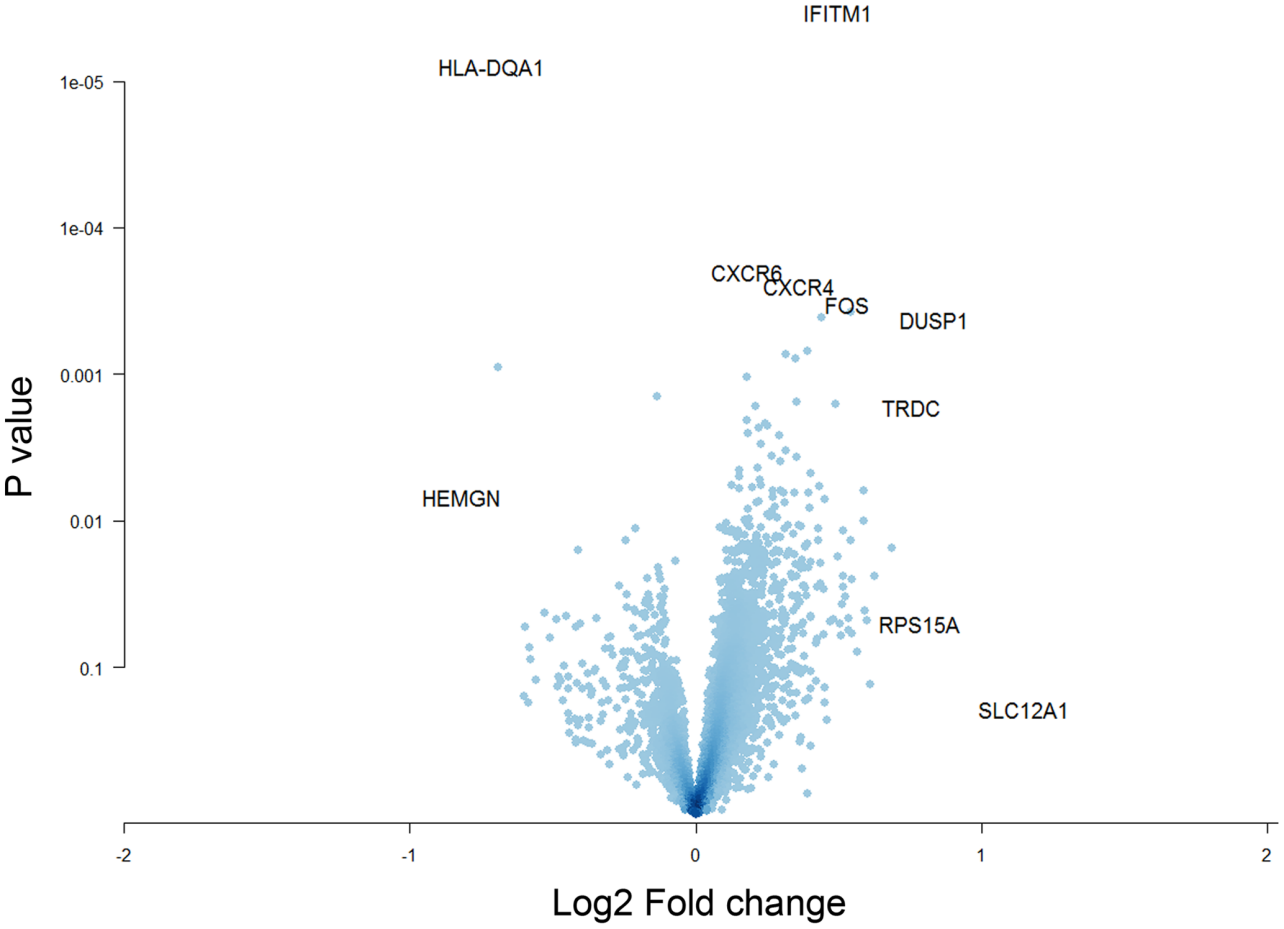
Identification of differentially expressed genes in PBL during acute anaphylaxis at ED arrival. Gene expression patterns in PBL from anaphylaxis patients and healthy controls were profiled on microarrays, and differentially expressed genes were identified with LIMMA. The data are plotted along axes of statistical significance and fold change (Volcano Plot).

Gene expression levels were initially compared between patients with acute anaphylaxis and healthy controls at each timepoint. At ED arrival, only two genes were differentially expressed; one gene was upregulated (Interferon-inducible transmembrane protein 1 (IFITM1)) during acute anaphylaxis in comparison to controls and the other gene was downregulated (HLA-DQA1, Fig. 1). At one hour post arrival, 67 genes were differentially expressed; 44 were upregulated and 23 were downregulated during anaphylaxis. Strikingly, at 3 hours post arrival, 2801 genes were differentially expressed; 1104 of which were upregulated and 1697 were downregulated.

### Differential expression of biological pathways during acute anaphylaxis

At ED arrival, not enough genes were differentially expressed for meaningful pathways analysis. However, a molecular signature derived from interferon-producing killer dendritic cells [21] (e.g. granzyme A (GZMA), granzyme B (GZMB), killer cell lectin-like receptor C1 (KLRC1), KLRD1, KLRG1, natural killer cell group 7 (NKG7), perforin (PRF1)) was upregulated (false discovery rate (FDR)<0.001).

At one hour post arrival, bioinformatics analyses identified activation signatures downstream of lipopolysaccharide (LPS), tumor necrosis factor (TNF), prostaglandin E2, and Interleukin-1B (IL-1B) stimulation, and drugs (dexamethasone, prednisolone, norepinephrine) (overlap p-value <1×10−8) (Table S1). T cell related pathways were downregulated in the response.

At 3 hours post arrival, upregulated genes were mainly involved in the inflammatory response (DAVID p = 8.3×10^−14^), activation of the mitogen-activated protein kinase (MAPK) cascade (DAVID p = 5.0×10^−9^), response to LPS (DAVID p = 2.1×10^−8^), innate immune response (DAVID p = 7.9×10^−7^), apoptosis/cell death (DAVID p = 1.8×10^−6^), organization of the actin cytoskeleton (DAVID p = 2.5×10^−6^) and chemotaxis (DAVID p = 4.0×10^−6^). Major inflammatory signaling pathways that were upregulated included toll like receptor (TLR), triggering receptor expressed on myeloid cells (TREM1), NFκB, and multiple cytokines (Interferon-γ (IFNγ), IL-1, IL-4, IL-6, IL-8, IL-10, transforming growth factor (TGFβ) and TNF) (Table S2). The downregulated genes were mainly involved in T cell signaling/activation, and the protein translation/synthesis machinery (Table S3). LPS and TNF were again the main upstream regulators.

### Differential expression of gene coexpression networks during acute anaphylaxis

**Figure 2:**
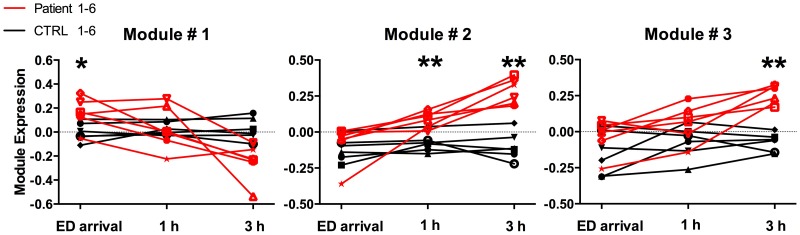
Time course of module gene expression in anaphylaxis patients and healthy controls. The expression pattern of each module was summarized using principal components analysis, and the first component was plotted. Statistical analysis by correlation adjusted gene set analysis. *p = 0.06, **p<0.01.

**Figure 3:**
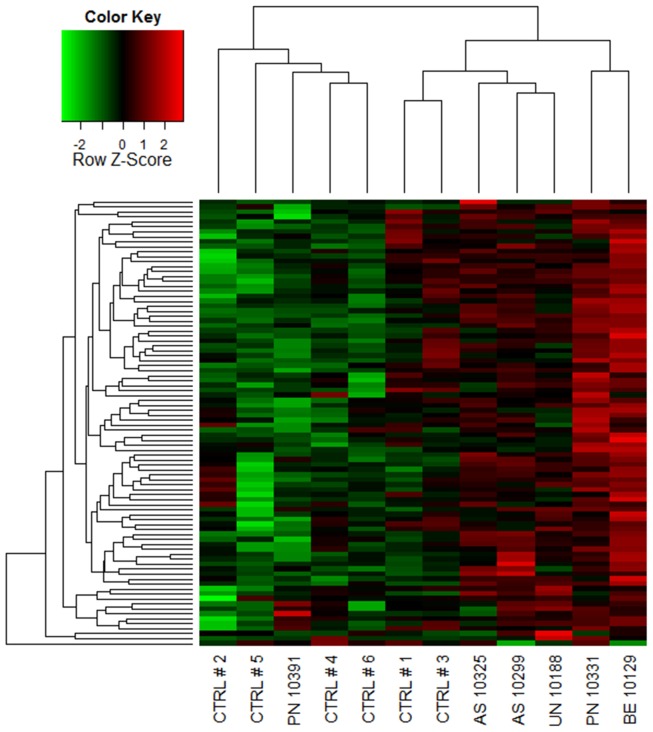
Module # 1 was upregulated at ED arrival in a subset of the anaphylaxis patients. Network analysis was employed to identify gene coexpression modules in PBL responses during acute anaphylaxis. The heatmap illustrates the expression of module # 1 at ED arrival for individual patients and controls. Hierarchical cluster analysis was employed to cluster genes and samples based on the similarity of their expression patterns. (PN – peanut anaphylaxis, AS – aspirin anaphylaxis, BE – bee anaphylaxis, UN – unknown anaphylaxis, CTRL – healthy control).

**Figure 4:**
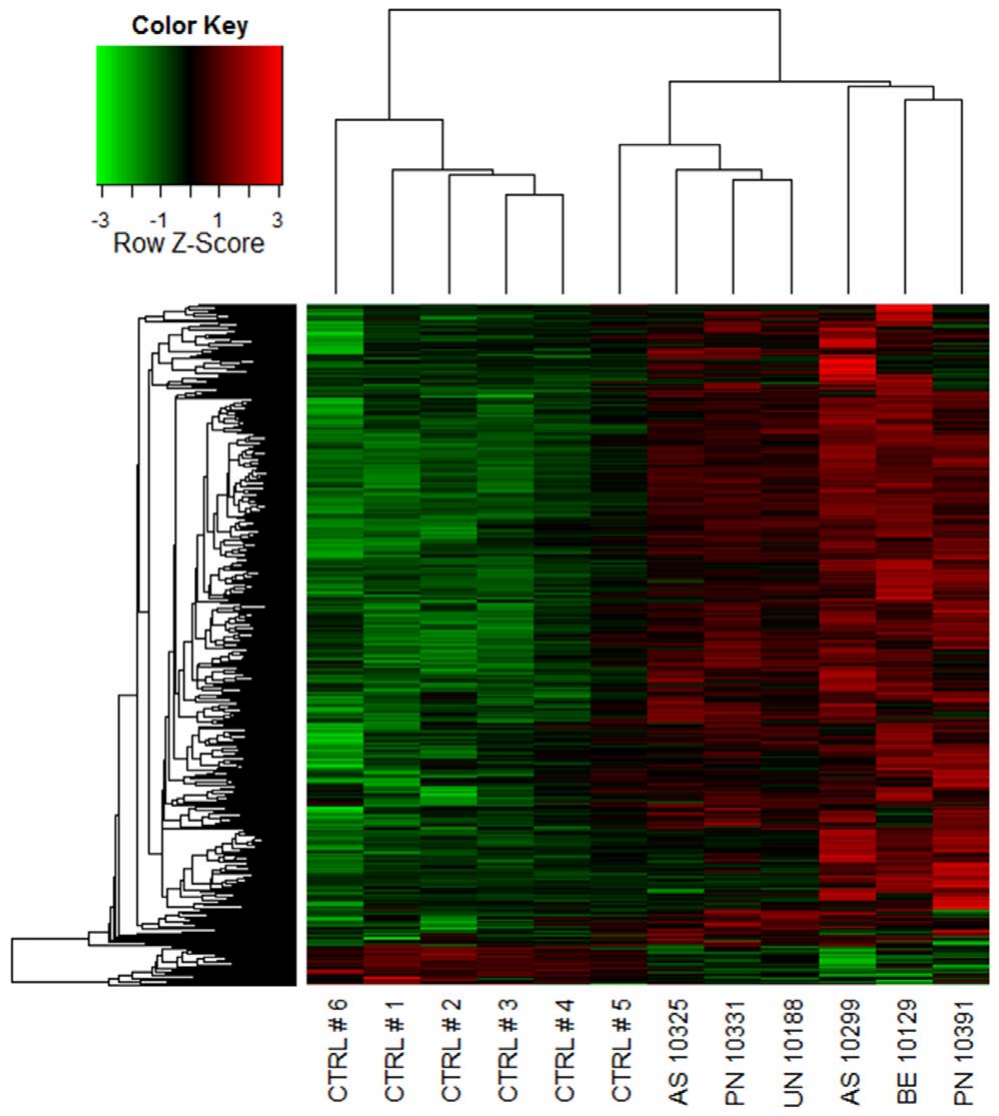
Module # 2 was upregulated at both 1 and 3 hours post ED arrival in anaphylaxis patients. Network analysis was employed to identify gene coexpression modules in PBL responses during acute anaphylaxis. The heatmap illustrates the expression of module # 2 at 3 hours post ED arrival for individual patients and controls. Hierarchical cluster analysis was employed to cluster genes and samples based on the similarity of their expression patterns. (PN – peanut anaphylaxis, AS – aspirin anaphylaxis, BE – bee anaphylaxis, UN – unknown anaphylaxis, CTRL – healthy control).

**Figure 5:**
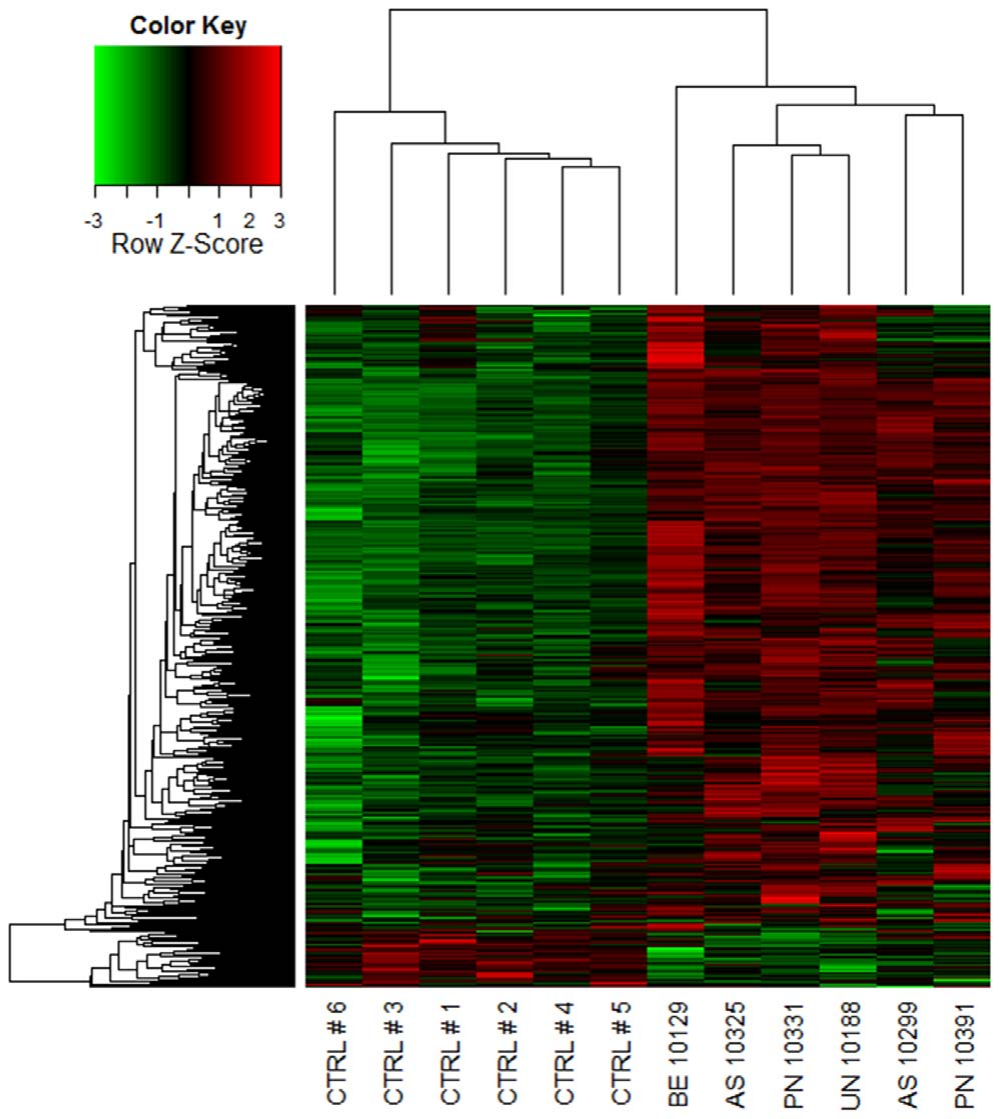
Module # 3 was upregulated at 3 hours post ED arrival in anaphylaxis patients. Network analysis was employed to identify gene coexpression modules in PBL responses during acute anaphylaxis. The heatmap illustrates the expression of module # 3 at 3 hours post ED arrival for individual patients and controls. Hierarchical cluster analysis was employed to cluster genes and samples based on the similarity of their expression patterns. (PN – peanut anaphylaxis, AS – aspirin anaphylaxis, BE – bee anaphylaxis, UN – unknown anaphylaxis, CTRL – healthy control).

A coexpression network was constructed to obtain a systems level view of the anaphylactic inflammatory response. This analysis utilized information gleaned from gene correlation patterns across the samples to elucidate the topology of the underlying gene networks. Gene networks are organized into smaller functional units of highly correlated genes known as modules, which carry out specific biological functions. Altered module behavior is thought to give rise to disease states [22]. The resulting coexpression network comprised 5,292 genes organized into 10 modules (data not shown). These modules were tested for differential expression in anaphylaxis cases versus controls at each individual time point, employing gene set analysis. This statistical method tests the association of a set of genes with a phenotype of interest, deriving a single p-value for the gene set [23]. At ED arrival, module #1 was upregulated, however this did not reach statistical significance (p = 0.06), because it was not consistently hyper-expressed across all of the patients (Fig. 2A, Fig. 3). Module #2 was significantly upregulated at 1 and 3 hours post ED arrival (Fig. 2B, Fig 4), and module #3 was also upregulated at 3 hours post ED arrival (Fig. 2C, Fig 5).

Module #1 was enriched for NK receptors and genes involved in cytotoxic functions (eomesodermin (EOMES), KLRC1, KLRD1, KLRG1, NKG7, granulysin, GZMA, GZMB, PRF1, IL-2RB, IL-12RB2). Upstream regulator analysis suggested that this module was driven by cytokines that promote Th1 and cytotoxic responses (IL-15, IL-2, IL-21, IL-12 overlap p<1×10^−16^−1×10^−9^) [24].

**Figure 6:**
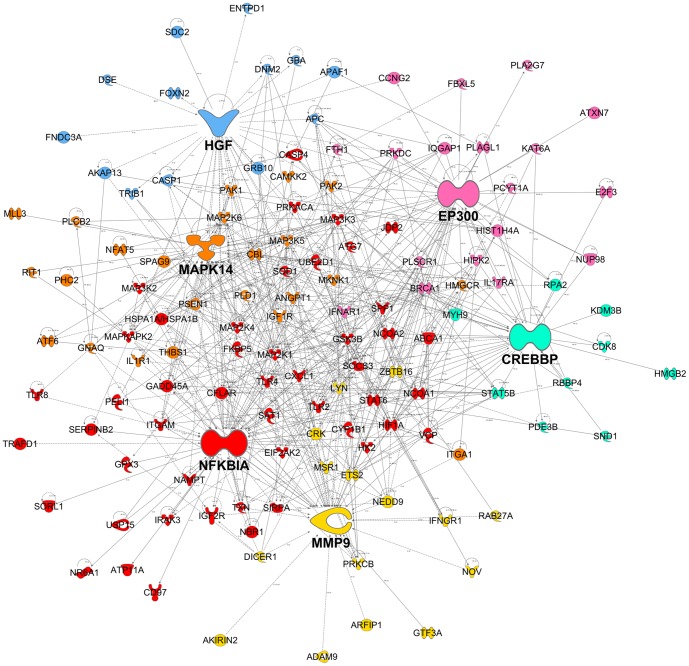
A reconstruction of module # 2. Mechanistic data from previous studies was utilized to build a molecular interaction network. For clarity, the figure illustrates the top six most interactive hubs and their neighbours. The genes are colour coded according to the hub that they are connected to.

Module #2 was enriched for genes involved in the MAPK cascade (MAPK14, MAP2K1, MAP2K4, MAP2K6, MAP3K2, MAP3K3, MAP3K5, MAP4K4, MAPKAPK2, DAVID p = 2.2×10^−6^), positive regulation of cell death (e.g. apoptotic peptidase activating factor 1 (APAF1), caspase recruitment domain family 6 (CARD6), caspase 1 (CASP1), CASP4, CASP8 and FADD-like apoptosis regulator (CFLAR), death-associated protein kinase 2 (DAPK2), DAVID p = 1.0×10^−5^), and the inflammatory response (e.g. chemokine (C-X-C motif) ligand 1 (CXCL1), complement component (3b/4b) receptor 1 (CR1), complement component 3a receptor (C3AR1), IL-1 receptor accessory protein (IL1RAP), IL18RAP, (thrombospondin) THBS1, (DAVID p = 1.3×10^−3^). Classical pathways identified in this module were NFκB signaling, TLR signaling (TLR2, TLR4, TLR8, IL-1 receptor-associated kinase (IRAK), MAPK, NFκB), p38 Map Kinase, IL-1 and IL-6 signaling (Table S4). Upstream regulator analysis demonstrated that LPS was the most prominent activation signature identified in Module #2 (overlap p<3.9×10^−12^). Other upstream regulators implicated in this module were transglutaminase 2 (TGM2), TNF, oncostatin M (OSM), tumor protein p53 (TP53), IFNγ, IL-4, IL-1B, and others. A reconstruction of Module #2 showing the top six hub genes is illustrated in Fig. 6. The top six hub genes in this module were nuclear factor of kappa light polypeptide gene enhancer in B-cells inhibitor alpha (NFKBIA; also known as IκBα), MAPK14 (also known as p38), matrix metallopeptidase-9 (MMP9), E1A binding protein p300 (EP300; also known as p300, KAT3B, RSTS2), hepatocyte growth factor (HGF; also known as hepapoietin A, scatter factor) and CREB binding protein (CREBBP; also known as CBP, RSTS, KAT3A).

**Figure 7:**
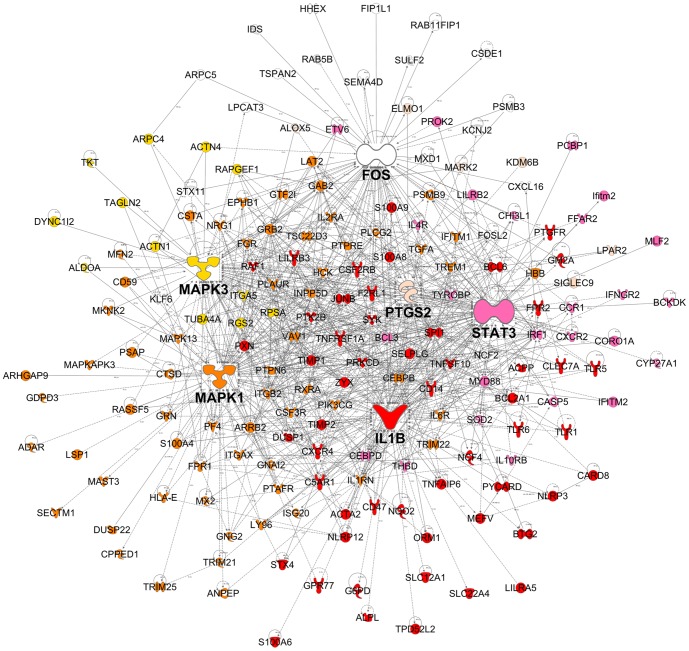
A reconstruction of module # 3. Mechanistic data from previous studies was utilized to build a molecular interaction network. For clarity, the figure illustrates the top six most interactive hubs and their neighbours. The genes are colour coded according to the hub that they are connected to.

Module #3 was enriched for genes involved in the inflammatory response (e.g. arachidonate 5-lipoxygenase (ALOX5), CCAAT/enhancer binding protein beta (CEBPB), IL1B, orosomucoid 1 (ORM1), NLR family, pyrin domain containing 3 (NLRP3), S100A8, S100A9, S100A12, DAVID p = 1.4×10^−16^), chemotaxis (e.g. CCR1, CXCR1, CXCR2, CXCR4, CXCL16, formyl peptide receptor 1 (FPR1), platelet factor 4 (PF4), DAVID p = 2.5×10^−9^), actin cytoskeleton organization (e.g. actinin 1 (ACTN1), ACTN4, actin related protein 2/3 complex subunit 1A (ARPC1A), ARPC5, DAVID p = 6.4×10^−6^), and programmed cell death (e.g. caspase recruitment domain family 8 (CARD8), caspase 5 (CASP5), cathepsin D (CTSD), death effector domain 2 (DEDD2), superoxide dismutase 2 (SOD2), TNR receptor 1 (TNF-RI), TNF-related apoptosis inducing ligand (TRAIL), DAVID p = 3.8×10^−5^). Classical pathways identified in this module were TREM1 signaling, leukocyte extravasation signaling, Fcγ receptor-mediated signaling, N-formylmethionyl-leucyl-phenylalanine (fMLP) signaling in neutrophils, acute phase response, IL-6 signaling, and TLR signaling (TLR1, TLR5, TLR6, CD14, MyD88) (Table S5). Upstream regulator analysis demonstrated that the most prominent activation signature in this module was also LPS (overlap p = 6.1×10^−30^). Other upstream regulators implicated in driving the response include TNF, IFNγ, TGFβ, CEBPA, TGM2, STAT3, TP53, IL-6, prostaglandin E2, IL-1β, IFNα and others. A reconstruction of Module #3 showing the top six hub genes is illustrated in Fig. 7. The top six hub genes in this module were IL-1β (IL-1B), MAPK1, signal transducer and activator of transcription 3 (STAT3; also known as acute-phase response factor), MAPK3 (also known as ERK-1), FBJ murine osteosarcoma viral oncogene homolog (FOS; also known as p55, AP1, C-FOS) and prostaglandin-endoperoxide synthase 2 (PTGS2; also known as COX2). A full list of hub genes (defined as at least five links) for Module #2 and #3 are available in Table S6.

## Discussion

We found that limited initial gene expression in PBL was followed by a striking upregulation of the innate immune response with a maximal response observed three hours after ED arrival, approximately four to six hours after reaction onset. Coexpression network analysis identified 3 functionally coherent modules that were upregulated at one or more time points during anaphylaxis:

- Module #1 was upregulated at ED arrival and was enriched for NK receptors and genes involved in cytotoxic function. A molecular signature for interferon-producing killer dendritic cells [21] was detected in module #1 and IFITM1 was significantly upregulated compared to controls at ED arrival (IFITM proteins are induced by Type I and II interferons).
- Module #2 was upregulated at both 1 and 3 hours post-arrival and was enriched for genes involved in the MAPK cascade, and positive regulation of cell death. Major hub genes in this module include CREBBP, EP300, HGF, MAPK14, MMP9, and NFKBIA.
- Module #3 was upregulated at 3 hours post-arrival and was enriched for genes involved in the inflammatory response and chemotaxis, including TREM1 signaling (myeloid cell activation) and leukocyte extravasation signaling. Major hub genes in this module were FOS, IL-1B, MAPK1, MAPK3, PTGS2, and STAT3.
- Bioinformatics analyses identified prominent LPS-like and TNF activation signatures in the data, with gene activation patterns similar to that seen during sepsis, including TLR-mediated responses.

Little is known about the role of innate immune responses during anaphylaxis. TLR-mediated responses were prominent in our analysis. These are known to be triggered by both pathogen-associated molecular patterns (PAMPs) and danger-associated molecular pattern (DAMPs) [25]. DAMPs include endogenous danger signals released from damaged and necrotic cells and alarmins such as granulosyins, defensins, lactoferrin, the S100 proteins and high mobility group box 1 (HMGB1) [26], [27]. In our study, module # 3 contained several members of the S100 family (S100A8, S100A9, S100A12), and HMGB1 and HMGB2 were also detected in the responses. S100A8 and S100A9 are endogenous TLR4 ligands that promote endotoxin-induced shock [28]. HMGB1 is a nuclear protein that is released or secreted following trauma or severe cellular stress and triggers inflammation and recruits leukocytes to the site of tissue damage [29]. HMGB1 can bind to TLR4 and the CXCR4 receptor when complexed with CXCL12 [30].

We observed upregulation of genes that regulate TLR responses (MyD88, MAPK) and TLR genes themselves (TLR1, TLR2, TLR4, TLR5, TLR6, TLR8). Both modules #2 and #3 identified upregulation of TLR signaling but differentiated between the specific TLRs triggered (i.e. module #2 TLR2, TLR4, TLR8 and module #3 TLR1, TLR5, TLR6). This difference may represent a shift in the concentration of specific activating/regulatory molecules over time or changes in the activation of specific populations of peripheral blood cells. For example, monocytes and neutrophils express all TLR family members except TLR3 and TLR7, with TLR2 and TLR4 most highly expressed on monocytes [31], [32], whereas plasmacytoid dendritic cells (pDC) do not express TLR1, TLR2, TLR3, TLR4, TLR5 or TLR6, but express TLR7 and 9 [33].

Activation of TLRs results in the production of a large set of NFκB-dependent proinflammatory cytokines and type I IFNs induced via IFN regulatory factors. The type 1 IFN system may be activated during anaphylaxis through cell damage releasing self-nucleic acids, forming complexes with cellular alarmins and other proteins which facilitate endocytosis by pDC and induction of type 1 IFNs via TLR7 and TLR9 signaling [34]–[36].

Immune activation by infectious agents results in a remarkable crosstalk occurring among different cell types, leading to the amplification and/or modulation of the ongoing innate immune response [37]. Mast cells produce TNFα in response to TLR4 engagement by LPS [38], and type I IFN and various chemokines in response to TLR3 engagement by double stranded RNA [39]. This results in the activation and chemotaxis of peripheral blood cells such as neutrophils, DCs, monocytes and natural NK cells [40]–[42]. Activated pDCs and NK cells are also a potential source of IFNα during anaphylaxis. Holtzman and coworkers have shown that type I IFN signaling upregulates expression of the high affinity IgE receptor on dendritic cells, suggesting that type I IFNs may augment IgE-dependent immune pathways [43], and trigger both mild/moderate and severe asthma exacerbations [24], [44].

We observed a striking upregulation of gene expression over the three hours following ED arrival, with only 2 genes differentially expressed on arrival in the ED, 67 genes at one hour later and 2,801 genes after three hours. The small number of differentially expressed genes on arrival may have been because the majority of the early immune response was occurring in tissues at this time.

After one hour, upregulated genes included those downstream from prostaglandin E2, IL-1B and TNF signaling. All of these immune mediators are produced by activated mast cells [45]–[47]. As patients received adrenaline and/or steroids, it is not surprising that genes downstream of these drug-signaling pathways were upregulated after one hour. At three hours post-ED arrival, patients were no longer experiencing clinical symptoms and were preparing to be discharged from the ED. By this time (4–6 hours after reaction onset) a large number of differentially expressed genes were evident. Similarly, Calvano *et al* found similar changes in PBL gene expression patterns for innate immune responses, peaked in human subjects 4–6 hours after bolus injection of bacterial endotoxin [48]. Many of the same genes identified by Calvano *et al*, were present in our network analysis, including genes that initiate (IL-1B, CEBP, CREBBP) and limit/resolve the immune response (NFKBIA, STAT3, SOCS3, IL-1RAP). We identified upregulation of major inflammatory pathways, including TLR and TREM1, suggesting early involvement of the innate immune system and neutrophil activation. This is consistent with mouse models indicating a pivotal role for neutrophils in the anaphylaxis as generators of platelet activating factor (PAF) [49]. Genes involved in apoptosis/cell death were also upregulated, possibly indicating the timely apoptosis and clearance of neutrophils and other PBL that is essential for resolution of an inflammatory response [50]. Unfortunately, we were unable to collect samples from our patient cohort beyond three hours to assess the timing of complete resolution of the immune response and return to baseline.

A number of major hub genes that both initiate and resolve inflammatory responses were identified in module #2 and module #3. Many of the identified hub genes interact or directly activate each other, activate overlapping groups of target genes and are engaged in both negative and positive crosstalk. For example, the NFκB signaling pathway induces IL-1B, MMP9 and IκBα and p38 MAPK upregulates genes coding for IL-1B and PTGS2 (COX-2). P38α is required for activation of the transcription factor CREB and it contributes to the induction of several genes, including those encoding chemokines, cytokines and regulators of extracellular matrix remodeling and cell adhesion [51]. CREBBP and EP300 are co-activators that assist with CREB-induced transcription, which is involved in cell proliferation, survival, apoptosis and the innate immune response. NFKBIA (IκBα) tightly regulates the activity of NFκB with the modulation of NFKBIA regarded as an anti-inflammatory and immunosuppressive mechanism in asthma [52]. The balanced activation of the p38MAPK-pathway and STAT3-mediated signal transduction is essential for both induction and propagation of the inflammatory macrophage response as well as for the control of the resolution phase, which is largely driven by IL-10 and sustained STAT3 activation [53]. STAT3 is activated through phosphorylation in response to various cytokines and growth factors including IFNs, HGF and IL-6. IL-1β plays a central role in innate immunity and has been shown to induce urticarial rashes in autoinflammatory diseases and play a role in bronchial asthma, contact hypersensitivity and atopic dermatitis [54].

Both MMP9 and HGF may be important for vascular repair after acute damage. Neutrophils are a potent source of MMP9, one of the matrix metalloproteinase family, which are major proteins involved in tissue remodeling. Gene expression and plasma concentrations of MMP9 have been shown to be significant higher in ischemic stroke patients compared to healthy controls [55], and in severe sepsis [56]. In a mouse model, TLR2 activation of neutrophils led to the release of MMP9, which was protective against experimentally-induced asthma [57]. HGF has been proposed as a modulator of cardiac tissue repair [58]. The expression of HGF and its secretion into the blood circulation are promoted during the early phase of myocardial infarction [59]. By promoting angiogenesis and inhibiting apoptosis, endogenous HGF may play an important role in cardioprotection as well as in the regeneration of endothelial cells and cardiomyocytes after myocardial infarction [60].

This study has a number of limitations that must be acknowledged. The number of patients studied was small and patients experienced moderately severe anaphylaxis with no cases of hypotension or hypoxia. The attributed causes were also heterogenous, including possible IgE- and non-IgE-triggered anaphylaxis, and the effect of emergency treatment on gene expression was not controlled for. Patients also differed in the time taken to arrive in the ED after reaction onset, although it should be noted that all patients were untreated at ED arrival and all presented with similar skin and respiratory features. Although the time course design of the study increased the statistical power of our analyses, a follow up study is required in a larger number of patients to determine how variations in gene network patterns differ in relation to variations in reaction triggers and reaction severity. The gene expression profiling data was based on a mixed cell population from peripheral blood, therefore variations in the cellular composition of the samples may potentially limit the precision of the analysis. Whilst the data showed evidence of a cytotoxic response (including type I IFNs) and neutrophil activation during human anaphylaxis, the exact populations of PBL involved requires confirmation by other techniques such as flow cytometry. Nevertheless, this exploratory analysis of gene expression patterns during human anaphylaxis indicates a major role for the innate immune system in disease pathogenesis, and the hub genes identified in this study represent logical candidates for follow-up in detailed mechanistic studies.

## Supporting Information

Table S1 Top 10 canonical pathways and upstream regulators associated with acute human anaphylaxis at one hour post ED arrival. Differentially expressed genes were identified and analyzed in Ingenuity Systems software. Due to the limited number of differentially expressed genes at this time point, up- and down- regulated genes were analyzed together. Upstream regulators are only included when the activation state was predicted from Ingenuity Systems. The activation state can only be predicted when the direction of the gene expression changes are consistent with prior studies. **↑** =  molecules associated with this pathway were mainly upregulated. **↓** =  molecules associated with this pathway were mainly downregulated.
(DOCX)

Table S2 Canonical pathways and upstream regulators associated with the genes that were upregulated during acute human anaphylaxis at three hours post ED arrival. Differentially expressed genes were identified and analyzed in Ingenuity Systems software. The analysis was restricted to the upregulated genes only. Upstream regulators are only included when the activation state was predicted from Ingenuity Systems. The activation state can only be predicted when the direction of the gene expression changes are consistent with prior studies.
(DOCX)

Table S3 Canonical pathways and upstream regulators associated with the genes that were downregulated during acute human anaphylaxis at three hours post ED arrival. Differentially expressed genes were identified and analyzed in Ingenuity Systems software. The analysis was restricted to the downregulated genes only. Upstream regulators are only included when the activation state was predicted from Ingenuity Systems. The activation state can only be predicted when the direction of the gene expression changes are consistent with prior studies.
(DOCX)

Table S4 Canonical pathways and upstream regulators associated with the genes in module # 2. Anaphylaxis-associated module # 2 was analyzed in Ingenuity Systems software. The module contains both up and down regulated genes. **↑** =  molecules associated with this pathway were mainly upregulated. Upstream regulators are only included when the activation state was predicted from Ingenuity Systems. The activation state can only be predicted when the direction of the gene expression changes are consistent with prior studies.
(DOCX)

Table S5 Canonical pathways and upstream regulators associated with the genes in module # 3. Anaphylaxis-associated module # 3 was analyzed in Ingenuity Systems software. The module contains both up and down regulated genes. **↑** =  molecules associated with this pathway were mainly upregulated. Upstream regulators are only included when the activation state was predicted from Ingenuity Systems. The activation state can only be predicted when the direction of the gene expression changes are consistent with prior studies.
(DOCX)

Table S6 Hub genes identified in Module #2 and Module #3.
(DOCX)
