# Supplementary material for: Genomic Responses during Acute Human Anaphylaxis Are Characterized by Upregulation of Innate Inflammatory Gene Networks
Source: PLoS One. 2014 Jul 1;9(7):e101409. doi: 10.1371/journal.pone.0101409 (PMC4077795; doi:10.1371/journal.pone.0101409)
Supplement: Table S1 — Top 10 canonical pathways and upstream regulators associated with acute human anaphylaxis at one hour post ED arrival. Differentially expressed genes were identified and analyzed in Ingenuity Systems software. Due to the limited number of differentially expressed genes at this time point, up- and down- regulated genes were analyzed together. Upstream regulators are only included when the activation state was predicted from Ingenuity Systems. The activation state can only be predicted when the direction of the gene expression changes are consistent with prior studies. ↑ = molecules associated with this pathway were mainly upregulated. ↓ = molecules associated with this pathway were mainly downregulated. (DOCX) [file pone.0101409.s001.docx]

Table S1: Top 10 canonical pathways and upstream regulators associated with acute human anaphylaxis at one hour post ED arrival.

| **#** | **Canonical Pathways** | **P-value** |  | **#** | **Upstream Regulator** | **P-value** |
| --- | --- | --- | --- | --- | --- | --- |
| **1** | **↓** Altered T Cell and B Cell Signaling in Rheumatoid Arthritis | 1.51E-03 |  | **1** | lipopolysaccharide | 5.82E-11 |
| **2** | **↓** Autoimmune Thyroid Disease Signaling | 2.45E-03 |  | **2** | norepinephrine | 3.47E-09 |
| **3** | **↑** NF-κB Signaling | 2.69E-03 |  | **3** | TNF | 9.34E-09 |
| **4** | **↓** Role of NFAT in Regulation of the Immune Response | 2.95E-03 |  | **4** | prostaglandin E2 | 1.05E-08 |
| **5** | **↓** iCOS-iCOSL Signaling in T Helper Cells | 3.31E-03 |  | **5** | prednisolone | 1.50E-08 |
| **6** | **↑** Toll-like Receptor Signaling | 4.07E-03 |  | **6** | dexamethasone | 1.75E-08 |
| **7** | **↑** CD40 Signaling | 5.62E-03 |  | **7** | forskolin | 1.83E-08 |
| **8** | **↑** IL-10 Signaling | 6.92E-03 |  | **8** | bucladesine | 2.02E-07 |
| **9** | **↓** T Helper Cell Differentiation | 7.24E-03 |  | **9** | IL1B | 2.03E-07 |
| **10** | **↑** Relaxin Signaling | 7.41E-03 |  | **10** | poly rI:rC-RNA | 3.78E-06 |

Differentially expressed genes were identified and analyzed in Ingenuity Systems software. Due to the limited number of differentially expressed genes at this time point, up- and down- regulated genes were analyzed together. Upstream regulators are only included when the activation state was predicted from Ingenuity Systems. The activation state can only be predicted when the direction of the gene expression changes are consistent with prior studies. **↑** = molecules associated with this pathway were mainly upregulated. **↓** = molecules associated with this pathway were mainly downregulated.
