# Supplementary material for: Genomic Responses during Acute Human Anaphylaxis Are Characterized by Upregulation of Innate Inflammatory Gene Networks
Source: PLoS One. 2014 Jul 1;9(7):e101409. doi: 10.1371/journal.pone.0101409 (PMC4077795; doi:10.1371/journal.pone.0101409)
Supplement: Table S2 — Canonical pathways and upstream regulators associated with the genes that were upregulated during acute human anaphylaxis at three hours post ED arrival. Differentially expressed genes were identified and analyzed in Ingenuity Systems software. The analysis was restricted to the upregulated genes only. Upstream regulators are only included when the activation state was predicted from Ingenuity Systems. The activation state can only be predicted when the direction of the gene expression changes are consistent with prior studies. (DOCX) [file pone.0101409.s002.docx]

Table S2: Canonical pathways and upstream regulators associated with the genes that were upregulated during acute human anaphylaxis at three hours post ED arrival.

| **#** | **Canonical Pathways** | **P-value** |  | **#** | **Upstream Regulator** | **P-value** |
| --- | --- | --- | --- | --- | --- | --- |
| **1** | Toll-like Receptor Signaling | 1.58E-11 |  | **1** | lipopolysaccharide | 1.75E-33 |
| **2** | IL-6 Signaling | 6.31E-11 |  | **2** | TNF | 1.14E-20 |
| **3** | GNRH Signaling | 1.41E-10 |  | **3** | TGM2 | 1.26E-17 |
| **4** | B Cell Receptor Signaling | 3.47E-10 |  | **4** | IFNG | 5.58E-16 |
| **5** | Role of Macrophages, Fibroblasts and Endothelial Cells in Rheumatoid Arthritis | 3.63E-10 |  | **5** | TGFB1 | 1.92E-15 |
| **6** | Molecular Mechanisms of Cancer | 6.92E-10 |  | **6** | IL4 | 6.57E-14 |
| **7** | IL-10 Signaling | 7.76E-10 |  | **7** | tretinoin | 1.93E-13 |
| **8** | IL-8 Signaling | 1.26E-09 |  | **8** | forskolin | 4.76E-13 |
| **9** | PPAR Signaling | 2.29E-09 |  | **9** | IL10 | 7.44E-13 |
| **10** | Fcγ Receptor-mediated Phagocytosis in Macrophages and Monocytes | 1.00E-08 |  | **10** | TP53 | 4.78E-12 |
| **11** | PPARα/RXRα Activation | 1.66E-08 |  | **11** | poly rI:rC-RNA | 9.38E-12 |
| **12** | FLT3 Signaling in Hematopoietic Progenitor Cells | 2.45E-08 |  | **12** | phorbol myristate acetate | 9.81E-12 |
| **13** | NF-κB Signaling | 8.51E-08 |  | **13** | OSM | 1.23E-11 |
| **14** | iNOS Signaling | 9.12E-08 |  | **14** | SELPLG | 2.04E-11 |
| **15** | Role of Pattern Recognition Receptors in Recognition of Bacteria and Viruses | 9.33E-08 |  | **15** | CEBPA | 2.91E-11 |
| **16** | Prolactin Signaling | 1.12E-07 |  | **16** | IL6 | 5.10E-11 |
| **17** | TREM1 Signaling | 1.35E-07 |  | **17** | IL1B | 1.40E-10 |
| **18** | Cardiac Hypertrophy Signaling | 1.45E-07 |  | **18** | CSF3 | 1.62E-10 |
| **19** | HGF Signaling | 1.62E-07 |  | **19** | IL13 | 1.69E-10 |
| **20** | IL-1 Signaling | 1.74E-07 |  | **20** | IL2 | 4.33E-10 |

Differentially expressed genes were identified and analyzed in Ingenuity Systems software. The analysis was restricted to the upregulated genes only. Upstream regulators are only included when the activation state was predicted from Ingenuity Systems. The activation state can only be predicted when the direction of the gene expression changes are consistent with prior studies.
