# Supplementary material for: Genomic Responses during Acute Human Anaphylaxis Are Characterized by Upregulation of Innate Inflammatory Gene Networks
Source: PLoS One. 2014 Jul 1;9(7):e101409. doi: 10.1371/journal.pone.0101409 (PMC4077795; doi:10.1371/journal.pone.0101409)
Supplement: Table S3 — Canonical pathways and upstream regulators associated with the genes that were downregulated during acute human anaphylaxis at three hours post ED arrival. Differentially expressed genes were identified and analyzed in Ingenuity Systems software. The analysis was restricted to the downregulated genes only. Upstream regulators are only included when the activation state was predicted from Ingenuity Systems. The activation state can only be predicted when the direction of the gene expression changes are consistent with prior studies. (DOCX) [file pone.0101409.s003.docx]

Table S3: Canonical pathways and upstream regulators associated with the genes that were downregulated during acute human anaphylaxis at three hours post ED arrival.

| **#** | **Canonical Pathways** | **P-value** |  | **#** | **Upstream Regulator** | **P-value** |
| --- | --- | --- | --- | --- | --- | --- |
| **1** | EIF2 Signaling | 5.01E-33 |  | **1** | 5-fluorouracil | 1.32E-23 |
| **2** | Regulation of eIF4 and p70S6K Signaling | 5.01E-15 |  | **2** | CD 437 | 2.12E-20 |
| **3** | iCOS-iCOSL Signaling in T Helper Cells | 1.00E-12 |  | **3** | sirolimus | 1.43E-11 |
| **4** | mTOR Signaling | 2.00E-11 |  | **4** | CD3 | 8.95E-11 |
| **5** | CD28 Signaling in T Helper Cells | 1.23E-09 |  | **5** | miR-16-5p (and other miRNAs w/seed AGCAGCA) | 4.39E-06 |
| **6** | PKCθ Signaling in T Lymphocytes | 5.25E-09 |  | **6** | CD28 | 1.91E-05 |
| **7** | T Cell Receptor Signaling | 5.50E-09 |  | **7** | alefacept | 2.39E-05 |
| **8** | Calcium-induced T Lymphocyte Apoptosis | 5.75E-09 |  | **8** | miR-1 (and other miRNAs w/seed GGAAUGU) | 3.13E-03 |
| **9** | Role of NFAT in Regulation of the Immune Response | 5.01E-08 |  | **9** | CUL4B | 7.41E-03 |
| **10** | CTLA4 Signaling in Cytotoxic T Lymphocytes | 1.48E-07 |  | **10** | tanespimycin | 1.45E-02 |

Differentially expressed genes were identified and analyzed in Ingenuity Systems software. The analysis was restricted to the downregulated genes only. Upstream regulators are only included when the activation state was predicted from Ingenuity Systems. The activation state can only be predicted when the direction of the gene expression changes are consistent with prior studies.
