# Supplementary material for: Genomic Responses during Acute Human Anaphylaxis Are Characterized by Upregulation of Innate Inflammatory Gene Networks
Source: PLoS One. 2014 Jul 1;9(7):e101409. doi: 10.1371/journal.pone.0101409 (PMC4077795; doi:10.1371/journal.pone.0101409)
Supplement: Table S4 — Canonical pathways and upstream regulators associated with the genes in module # 2. Anaphylaxis-associated module # 2 was analyzed in Ingenuity Systems software. The module contains both up and down regulated genes. ↑ = molecules associated with this pathway were mainly upregulated. Upstream regulators are only included when the activation state was predicted from Ingenuity Systems. The activation state can only be predicted when the direction of the gene expression changes are consistent with prior studies. (DOCX) [file pone.0101409.s004.docx]

Table S4: Canonical pathways and upstream regulators associated with the genes in module # 2.

| **#** | **Canonical Pathways** | **P-value** |  | **#** | **Upstream Regulator** | **P-value** |
| --- | --- | --- | --- | --- | --- | --- |
| **1** | **↑** GNRH Signaling | 6.61E-08 |  | **1** | lipopolysaccharide | 3.98E-12 |
| **2** | **↑** PPARα/RXRα Activation | 1.17E-07 |  | **2** | TGM2 | 6.62E-10 |
| **3** | **↑** Molecular Mechanisms of Cancer | 5.75E-07 |  | **3** | OSM | 9.82E-09 |
| **4** | **↑** NF-κB Signaling | 4.27E-06 |  | **4** | TP53 | 1.06E-08 |
| **5** | **↑** Role of Macrophages, Fibroblasts and Endothelial Cells in Rheumatoid Arthritis | 1.38E-05 |  | **5** | TNF | 1.34E-08 |
| **6** | **↑** p53 Signaling | 1.55E-05 |  | **6** | IFNG | 9.31E-08 |
| **7** | **↑** Toll-like Receptor Signaling | 1.70E-05 |  | **7** | IL4 | 1.59E-06 |
| **8** | **↑** p38 MAPK Signaling | 4.37E-05 |  | **8** | IL1B | 2.32E-06 |
| **9** | **↑** PPAR Signaling | 8.13E-05 |  | **9** | APP | 4.40E-06 |
| **10** | **↑** IL-10 Signaling | 9.55E-05 |  | **10** | TGFB1 | 9.96E-06 |
| **11** | **↑** B Cell Receptor Signaling | 1.00E-04 |  | **11** | CSF3 | 1.01E-05 |
| **12** | **↑** FGF Signaling | 1.58E-04 |  | **12** | forskolin | 1.14E-05 |
| **13** | **↑** IL-6 Signaling | 1.62E-04 |  | **13** | beta-estradiol | 1.32E-05 |
| **14** | **↑** FLT3 Signaling in Hematopoietic Progenitor Cells | 1.95E-04 |  | **14** | lactacystin | 1.40E-05 |
| **15** | **↑** Pyridoxal 5'-phosphate Salvage Pathway | 2.34E-04 |  | **15** | phorbol myristate acetate | 2.10E-05 |
| **16** | **↑** CD27 Signaling in Lymphocytes | 3.02E-04 |  | **16** | TNFSF11 | 2.14E-05 |
| **17** | **↑** Type I Diabetes Mellitus Signaling | 3.09E-04 |  | **17** | dexamethasone | 3.04E-05 |
| **18** | **↑** Hypoxia Signaling in the Cardiovascular System | 3.39E-04 |  | **18** | IL1 | 3.32E-05 |
| **19** | **↑** Role of Pattern Recognition Receptors in Recognition of Bacteria and Viruses | 4.17E-04 |  | **19** | metribolone | 3.35E-05 |
| **20** | **↑** Role of IL-17A in Arthritis | 4.47E-04 |  | **20** | carbonyl cyanide m-chlorophenyl hydrazone | 3.77E-05 |

Anaphylaxis-associated module # 2 was analyzed in Ingenuity Systems software. The module contains both up and down regulated genes. **↑** = molecules associated with this pathway were mainly upregulated. Upstream regulators are only included when the activation state was predicted from Ingenuity Systems. The activation state can only be predicted when the direction of the gene expression changes are consistent with prior studies.
