# Supplementary material for: Genomic Responses during Acute Human Anaphylaxis Are Characterized by Upregulation of Innate Inflammatory Gene Networks
Source: PLoS One. 2014 Jul 1;9(7):e101409. doi: 10.1371/journal.pone.0101409 (PMC4077795; doi:10.1371/journal.pone.0101409)
Supplement: Table S5 — Canonical pathways and upstream regulators associated with the genes in module # 3. Anaphylaxis-associated module # 3 was analyzed in Ingenuity Systems software. The module contains both up and down regulated genes. ↑ = molecules associated with this pathway were mainly upregulated. Upstream regulators are only included when the activation state was predicted from Ingenuity Systems. The activation state can only be predicted when the direction of the gene expression changes are consistent with prior studies. (DOCX) [file pone.0101409.s005.docx]

Table S5: Canonical pathways and upstream regulators associated with the genes in module # 3.

| **#** | **Canonical Pathways** | **P-value** |  | **#** | **Upstream Regulator** | **P-value** |
| --- | --- | --- | --- | --- | --- | --- |
| **1** | **↑** TREM1 Signaling | 5.01E-11 |  | **1** | lipopolysaccharide | 6.11E-30 |
| **2** | **↑** Leukocyte Extravasation Signaling | 1.82E-10 |  | **2** | TNF | 9.25E-18 |
| **3** | **↑** Fcγ Receptor-mediated Phagocytosis in Macrophages and Monocytes | 1.12E-09 |  | **3** | IFNG | 7.51E-14 |
| **4** | **↑** Integrin Signaling | 3.02E-08 |  | **4** | TGFB1 | 1.42E-12 |
| **5** | **↑** IL-3 Signaling | 6.31E-08 |  | **5** | tretinoin | 2.77E-12 |
| **6** | **↑** fMLP Signaling in Neutrophils | 8.32E-08 |  | **6** | poly rI:rC-RNA | 3.37E-12 |
| **7** | **↑** Acute Phase Response Signaling | 1.41E-07 |  | **7** | CEBPA | 8.70E-12 |
| **8** | **↑** Ephrin Receptor Signaling | 2.57E-07 |  | **8** | camptothecin | 2.56E-11 |
| **9** | **↑** Remodeling of Epithelial Adherens Junctions | 2.75E-07 |  | **9** | dexamethasone | 4.49E-10 |
| **10** | **↑** Chemokine Signaling | 3.31E-07 |  | **10** | TGM2 | 9.89E-10 |
| **11** | **↑** Production of Nitric Oxide and Reactive Oxygen Species in Macrophages | 1.86E-06 |  | **11** | SELPLG | 1.45E-09 |
| **12** | **↑** IL-8 Signaling | 2.88E-06 |  | **12** | peptidoglycan | 2.88E-09 |
| **13** | **↑** Role of Pattern Recognition Receptors in Recognition of Bacteria and Viruses | 2.95E-06 |  | **13** | STAT3 | 7.95E-09 |
| **14** | **↑** Clathrin-mediated Endocytosis Signaling | 3.09E-06 |  | **14** | forskolin | 8.94E-09 |
| **15** | **↑** B Cell Receptor Signaling | 4.57E-06 |  | **15** | SPI1 | 1.62E-08 |
| **16** | **↑** IL-6 Signaling | 6.03E-06 |  | **16** | phorbol myristate acetate | 1.64E-08 |
| **17** | **↑** Actin Cytoskeleton Signaling | 7.76E-06 |  | **17** | SELP | 4.52E-08 |
| **18** | **↑** IL-12 Signaling and Production in Macrophages | 8.51E-06 |  | **18** | CpG oligonucleotide | 4.56E-08 |
| **19** | **↑** Thrombopoietin Signaling | 1.05E-05 |  | **19** | TP53 | 6.32E-08 |
| **20** | **↑** PI3K Signaling in B Lymphocytes | 2.04E-05 |  | **20** | F2 | 6.36E-08 |

Anaphylaxis-associated module # 3 was analyzed in Ingenuity Systems software. The module contains both up and down regulated genes. **↑** = molecules associated with this pathway were mainly upregulated. Upstream regulators are only included when the activation state was predicted from Ingenuity Systems. The activation state can only be predicted when the direction of the gene expression changes are consistent with prior studies.
