# Supplementary material for: Genomic Responses during Acute Human Anaphylaxis Are Characterized by Upregulation of Innate Inflammatory Gene Networks
Source: PLoS One. 2014 Jul 1;9(7):e101409. doi: 10.1371/journal.pone.0101409 (PMC4077795; doi:10.1371/journal.pone.0101409)
Supplement: Table S6 — Hub genes identified in Module #2 and Module #3. (DOCX) [file pone.0101409.s006.docx]

Table S6: Hub genes identified in Module #2 and Module #3

| **Module # 3** | | | **Module # 2** | | |
| --- | --- | --- | --- | --- | --- |
| **#** | **GeneID** | **Number of connections** | **#** | **GeneID** | **Number of connections** |
| 1 | IL1B | 91 | 1 | NFKBIA | 51 |
| 2 | MAPK1 | 80 | 2 | MAPK14 | 48 |
| 3 | STAT3 | 65 | 3 | MMP9 | 40 |
| 4 | MAPK3 | 64 | 4 | EP300 | 37 |
| 5 | FOS | 62 | 5 | HGF | 36 |
| 6 | PTGS2 | 45 | 6 | CREBBP | 33 |
| 7 | GRB2 | 38 | 7 | BRCA1 | 31 |
| 8 | CEBPB | 35 | 8 | GSK3B | 31 |
| 9 | RAF1 | 33 | 9 | HIF1A | 30 |
| 10 | ARRB2 | 32 | 10 | LYN | 30 |
| 11 | ITGB2 | 30 | 11 | MAP2K1 | 30 |
| 12 | MYD88 | 30 | 12 | PSEN1 | 29 |
| 13 | PTPN6 | 30 | 13 | TLR4 | 29 |
| 14 | SYK | 30 | 14 | SOCS3 | 25 |
| 15 | PRKCD | 26 | 15 | STAT6 | 24 |
| 16 | PXN | 26 | 16 | IGF1R | 21 |
| 17 | SPI1 | 26 | 17 | TLR2 | 21 |
| 18 | HCK | 25 | 18 | CBL | 19 |
| 19 | IRF1 | 25 | 19 | EIF2AK2 | 19 |
| 20 | VAV1 | 25 | 20 | SPP1 | 19 |
| 21 | CD14 | 25 | 21 | KIT | 18 |
| 22 | IL1RN | 24 | 22 | CRK | 17 |
| 23 | INPP5D | 23 | 23 | MAP2K6 | 17 |
| 24 | JUNB | 23 | 24 | MAP3K3 | 17 |
| 25 | PTK2B | 22 | 25 | MAP3K5 | 17 |
| 26 | CXCR4 | 20 | 26 | ETS2 | 15 |
| 27 | TIMP1 | 20 | 27 | HIST1H4A | 15 |
| 28 | GAB2 | 19 | 28 | MAP2K4 | 15 |
| 29 | TNFRSF1A | 19 | 29 | APC | 14 |
| 30 | TREM1 | 19 | 30 | GADD45A | 14 |
| 31 | NRG1 | 18 | 31 | IFNAR1 | 14 |
| 32 | PLAUR | 18 | 32 | PRKACA | 14 |
| 33 | SIRT7 | 18 | 33 | SOD1 | 14 |
| 34 | TNFSF10 | 18 | 34 | CXCL1 | 13 |
| 35 | PLCG2 | 17 | 35 | ERN1 | 13 |
| 36 | CEBPD | 16 | 36 | HSPA1A/HSPA1B | 13 |
| 37 | GNAI2 | 16 | 37 | MAPKAPK2 | 13 |
| 38 | IL2RA | 16 | 38 | PAK1 | 13 |
| 39 | ITGA5 | 15 | 39 | STAT5B | 13 |
| 40 | RXRA | 15 | 40 | DICER1 | 12 |
| 41 | BCL6 | 14 | 41 | VCP | 12 |
| 42 | MAPK13 | 14 | 42 | ACOX1 | 11 |
| 43 | SELPLG | 14 | 43 | IRS2 | 11 |
| 44 | DUSP1 | 13 | 44 | KDM5B | 11 |
| 45 | PF4 | 13 | 45 | PRKCB | 11 |
| 46 | CSF2RB | 12 | 46 | PRKDC | 11 |
| 47 | FGR | 12 | 47 | THBS1 | 11 |
| 48 | PIK3CG | 12 | 48 | ZBTB16 | 11 |
| 49 | SOD2 | 12 | 49 | CFLAR | 10 |
| 50 | TUBA1A | 12 | 50 | ITGAM | 10 |
| 51 | BCL3 | 11 | 51 | NCOA1 | 10 |
| 52 | C5AR1 | 11 | 52 | NCOA2 | 10 |
| 53 | CSF3R | 11 | 53 | NEDD9 | 10 |
| 54 | IL6R | 11 | 54 | PLSCR1 | 10 |
| 55 | TGFA | 11 | 55 | TXN | 10 |
| 56 | WAS | 11 | 56 | ABCA1 | 9 |
| 57 | GSN | 10 | 57 | CASP1 | 9 |
| 58 | LILRB3 | 10 | 58 | GNAQ | 9 |
| 59 | ACTN1 | 9 | 59 | IQGAP1 | 9 |
| 60 | BCL2A1 | 9 | 60 | mir-21 | 9 |
| 61 | EPHB1 | 9 | 61 | VAMP2 | 9 |
| 62 | FPR1 | 9 | 62 | ASAP1 | 8 |
| 63 | NCF2 | 9 | 63 | ATG7 | 8 |
| 64 | CXCR1 | 8 | 64 | HIPK2 | 8 |
| 65 | FLI1 | 8 | 65 | HMGCR | 8 |
| 66 | IL4R | 8 | 66 | MAP3K2 | 8 |
| 67 | PAF1 | 8 | 67 | RBBP4 | 8 |
| 68 | RPSA | 8 | 68 | UBE2D1 | 8 |
| 69 | TUBA4A | 8 | 69 | ATXN1 | 7 |
| 70 | CXCR1 | 7 | 70 | CASP4 | 7 |
| 71 | F2RL1 | 7 | 71 | IL1R1 | 7 |
| 72 | FOSL2 | 7 | 72 | MYH9 | 7 |
| 73 | GNG2 | 7 | 73 | PAK2 | 7 |
| 74 | HBB | 7 | 74 | PLAGL1 | 7 |
| 75 | ITGAX | 7 | 75 | PRDM5 | 7 |
| 76 | LAT2 | 7 | 76 | PTPRJ | 7 |
| 77 | NLRP12 | 7 | 77 | SAMSN1 | 7 |
| 78 | POR | 7 | 78 | SIRPA | 7 |
| 79 | PRMT5 | 7 | 79 | ADRB2 | 6 |
| 80 | PTAFR | 7 | 80 | FKBP5 | 6 |
| 81 | RASSF5 | 7 | 81 | MME | 6 |
| 82 | S100A4 | 7 | 82 | MSR1 | 6 |
| 83 | S100A8 | 7 | 83 | PLD1 | 6 |
| 84 | THBD | 7 | 84 | ANGPT1 | 5 |
| 85 | TIMP2 | 7 | 85 | ATF6 | 5 |
| 86 | TSC22D3 | 7 | 86 | CYP1B1 | 5 |
| 87 | TYROBP | 7 | 87 | DNM2 | 5 |
| 88 | ZYX | 7 | 88 | FTH1 | 5 |
| 89 | CCR1 | 7 | 89 | IL1RAP | 5 |
| 90 | ACTA2 | 6 | 90 | ITGA1 | 5 |
| 91 | ACTN4 | 6 | 91 | NBR1 | 5 |
| 92 | ARPC4 | 6 | 92 | RPA2 | 5 |
| 93 | CCND3 | 6 | 93 | SDC2 | 5 |
| 94 | CXCR2 | 6 | 94 | SLA | 5 |
| 95 | FPR2 | 6 | 95 | SND1 | 5 |
| 96 | GABARAP | 6 | 96 | VAMP3 | 5 |
| 97 | GRN | 6 | 97 | AKAP13 | 5 |
| 98 | NCF4 | 6 | 98 | CEACAM1 | 5 |
| 99 | NFYC | 6 | 99 | CSF2RA | 5 |
| 100 | RAPGEF1 | 6 | 100 | FLT3 | 5 |
| 101 | TNFSF13B | 6 | 101 | GRB10 | 5 |
| 102 | CD59 | 5 | 102 | IGF2R | 5 |
| 103 | CLEC7A | 5 | 103 | IL17RA | 5 |
| 104 | CSTA | 5 | 104 | MKNK1 | 5 |
| 105 | CTSD | 5 |  |  |  |
| 106 | DYSF | 5 |  |  |  |
| 107 | ETV6 | 5 |  |  |  |
| 108 | GABARAPL1 | 5 |  |  |  |
| 109 | GTF2I | 5 |  |  |  |
| 110 | IFITM1 | 5 |  |  |  |
| 111 | LY96 | 5 |  |  |  |
| 112 | NUMB | 5 |  |  |  |
| 113 | PSMB9 | 5 |  |  |  |
| 114 | S100A9 | 5 |  |  |  |
| 115 | SERPINA1 | 5 |  |  |  |
| 116 | CD47 | 5 |  |  |  |
| 117 | CHI3L1 | 5 |  |  |  |
| 118 | PAG1 | 5 |  |  |  |
| 119 | PTPRE | 5 |  |  |  |
| 120 | PYCARD | 5 |  |  |  |
| 121 | RGS2 | 5 |  |  |  |
